# Consecutive Eyeball Pressure Tests Reflect Clinically Relevant Vagal Dysfunction and Recovery in a Patient With Guillain-Barré-Syndrome With Tenacious Cardiac Dysautonomia

**DOI:** 10.3389/fneur.2020.483653

**Published:** 2020-09-29

**Authors:** Anouck Becker, Stefanie Behnke, Silke Walter, Mathias Fousse, Axel Buob, Jan Bürmann, Klaus Faßbender, Marcus M. Unger

**Affiliations:** ^1^Department of Neurology, Saarland University, Homburg, Germany; ^2^School of Medicine, Anglia Ruskin University, Chelmsford, United Kingdom; ^3^Department of Internal Medicine III, Saarland University, Homburg, Germany

**Keywords:** neuroimmunology, dysautonomia, Guillain-Barré-syndrome (GBS), Vagal dysfunction, mycoplasma pnemoniae, eyeball pressure testing, cardiac dysautonomia

## Abstract

Cardiac dysautonomia is a potentially life-threatening complication of Guillain-Barré syndrome (GBS). Proper and prompt recognition of patients at risk and subsequent intensive care unit (ICU) monitoring are mandatory to prevent fatal outcome. Eyeball pressure testing (EP) has been suggested as an easy applicable bedside test for vagal overreactivity in GBS and thus identifying patients at risk. Yet, there is only sparse follow-up data concerning the course of EP findings in GBS. We report a 25 years-old male patient with GBS who underwent consecutive EP (*n* = 11) during his ICU stay over a period of 11 weeks. The series of tests performed in this patient (and corresponding clinical events) show that EP data might represent an approximation of vagal dysfunction and vagal recovery in GBS. Interestingly, we observed a much longer duration of pathological EP compared to a previous report. The tenacious cardiac dysautonomia in this patient necessitated long-term application of a transvenous temporary pacemaker.

## Introduction

Cardiac dysautonomia is one of the major causes for mortality in GBS (1), stressing the need to identify and monitor patients at risk. Tetraplegic and artificially ventilated patients are generally considered to be particularly at risk (2, 3). Nevertheless, severe dysautonomic events can occur also in clinically less affected patients (4). Most frequent is the occurrence of sustained sinus tachycardia followed by brady- and tachyarrhythmias of atrial and ventricular origin (5). The cause of cardiac dysautonomia is still subject of investigation. Different hypotheses have been raised. Similar to demyelination of peripheral sensory and motor nerves, demyelination of autonomic afferent nerves from the heart are considered as cause of autonomic cardiac dysfunction. In severe cases, respiratory dysfunction might lead to secondary cardiac arrhythmia (6). Moreover, direct myocardial infiltration by inflammatory cells has been reported very early in context with GBS (7). Myocardial involvement, acute coronary syndromes and electrocardiographic changes without clinical correlate have also been reported (5). Vagal overreactivity as dreaded type of dysautonomia has been reported to last between 4 and 10 days (8). Standardized autonomic function tests such as Valsalva maneuvers, carotid pressure, short- and long-term heart rate variability have not proven to be successful in reflecting the risk of bradyarrhythmic events (5, 9). A certain prognostic value of blood pressure (BP) variation higher than 85 mmHg/day has been reported (3, 10), yet this elaborate way is rarely clinically practiced. Eyeball pressure testing (EP) has been reported to reflect vagal overreactivity and the risk of sudden bradyarrhythmia and asystole in GBS (8). Complications of EP are most frequently bradycardia and, rarely, retinal damage (8). There is no explicit data on the risk for retinal damage. Given the short duration (< 30 s) and haptic feedback concerning the extent of the applied pressure, damage of the retina is unlikely and has not yet occured in our own clinical experience.

## Case Report

A 25-year-old male patient was admitted to our emergency room due to a rapidly progressive flaccid tetraparesis and aphagia. He had had fever with dry cough starting 8 days prior to admission. Two days prior to admission, the patient noticed dysaesthesia of hands and feet. One day prior to admission, the patient noticed progressive weakness of all extremities. Past medical history was unremarkable.

Neurological examination on admission revealed a right-sided ptosis, dysphonia, aphagia with drooling, areflexia, a mainly left-sided flaccid tetraparesis and dysaesthesia of all extremities. The patient was orthopnoeic and breath sounds were decreased on the right side.

Due to the rapid progression of motor impairments and impending respiratory failure, the patient was transferred to our intensive care unit. Respiratory failure necessitated oral intubation on the day of admission.

Examination of the cerebrospinal fluid (CSF) on day 1 was unobtrusive, a re-puncture on day 14 showed an albuminocytologic dissociation (Table 1). Cerebral magnetic-resonance imaging (MRI) was normal. Chest x-ray showed right-sided perihilar pneumonia. Nerve conduction studies showed a demyelinating process. Laboratory testing ruled out differential diagnoses of acute demyelinating neuropathy other than GBS. Mycoplasma pneumoniae was identified as the cause for pneumonia and was successfully treated with meropeneme and roxithromycine. GBS was treated with 3 cycles of intravenous immunoglobulins (IVIG; 0.4 g per kilogram bodyweight for five consecutive days). IVIG therapy was initiated on the day of admission and was repeated on day 8 and again on day 46. IVIG therapy only led to a partial improvement of bulbar symptoms. Due to the severe clinical course, tracheostomy was performed and a percutaneous tube for parenteral nutrition was installed.

**Table 1:** Shows main cerebrospinal fluid findings.

**CSF results**	**Day of admission**	**Day 14 after admission**	**Day 35 after admission**
*Color*	Clear	Yellow	Yellow
*Cell count per μl*	3	7	20
*Protein in mg/dl*	39	2,500	582

**Figure 1:**
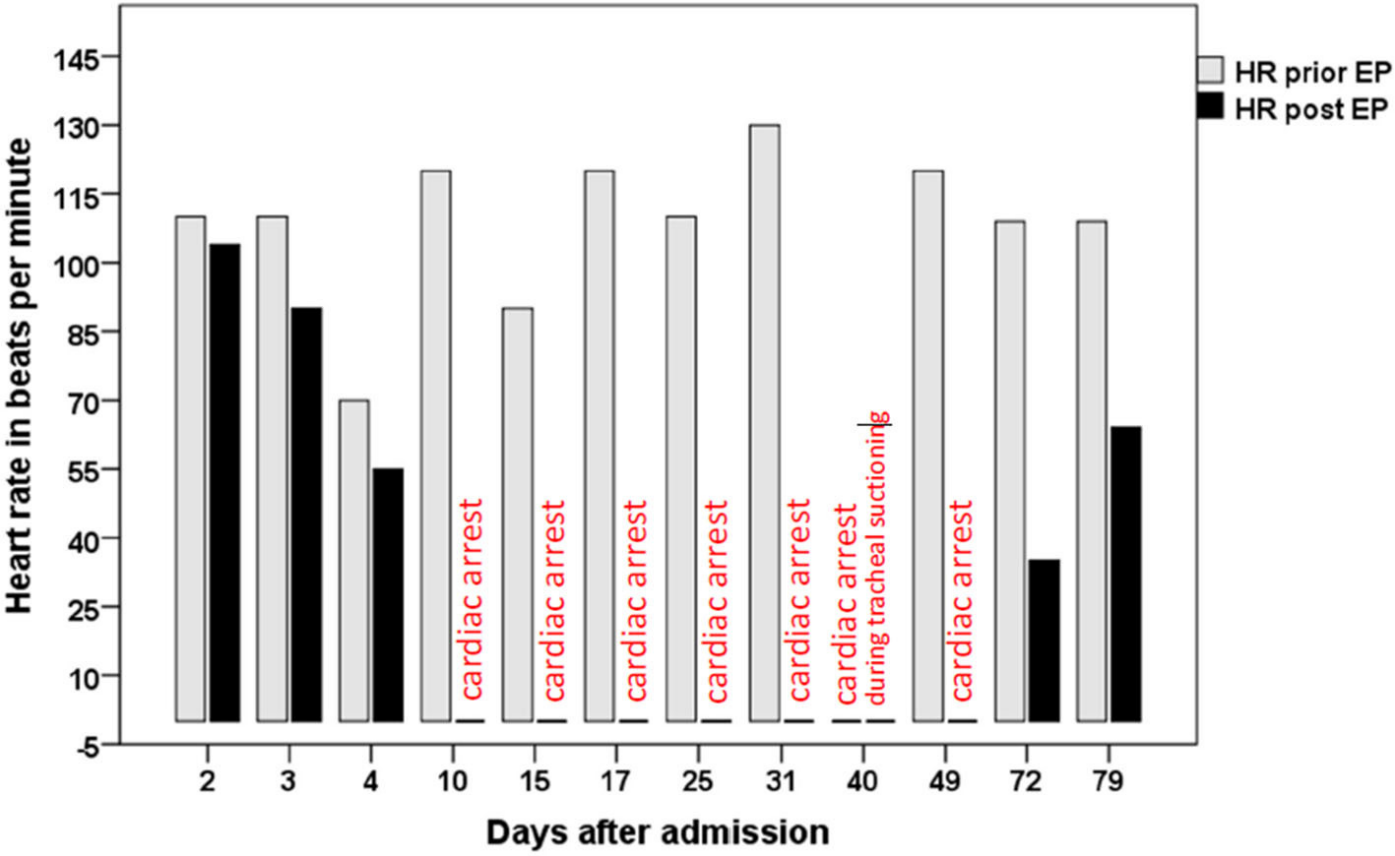
shows the heart rate in beats per minute prior (gray bars) and immediately after eyeball pressure testing (black bars, annotation “cardiac arrest,” respectively), at the 11 investigated points in time. On day 40, cardiac arrest occurred independently of EP during tracheal suctioning.

EP was performed (as described previously by Flachenecker and colleagues) on day 2, 3, 4, 10, 15, 17, 25, 31, 49, 72, and 79 after admission (Figure 1). EP on day 2 was normal, EP on day 3 and day 4 was pathological with a drop in heart rate of 110–90 bpm, 70–55 bpm, respectively. EP on day 10 resulted in a cardiac arrest that resolved spontaneously. Remarkably, the patient had shown no signs or symptoms of dysautonomia until then. Pathological EP prompted placement of a temporary transvenous pacemaker (PM) system with an active fixation bipolar lead, implanted through the subclavian access route. Due to persistent pathological EP and for prevention of infectious complications, the entry side and the lead were changed on day 33 and 57. Cardiac arrest (and consecutive pacing) occurred once during tracheal suctioning on day 40. On day 41, pacing was induced by bradycardia (< 50 bpm) during tracheal suctioning. On day 68 the PM lead dislocated and was removed without replacement.

On day 72 and day 79, EP still was pathological, but no more cardiac arrest was observed. On day 79, bradycardia was less pronounced compared to day 72. On day 81, the patient was transferred to a rehabilitation clinic. At this time, the patient was not able to speak but could communicate by facial mime and minimal voluntary movements of the head and jaw. Flaccid tetraplegia with areflexia persisted.

At a follow-up visit 20 months after the initial presentation, cranial nerve impairments and anarthria had resolved completely. While paresis of the upper extremities had mainly resolved, there were still major motor impairments of the lower extremities preventing the patient from walking independently. No cardiac events had occurred since discharge from our hospital.

## Discussion

The presented case is paradigmatic for a severe course of GBS. The severity and rapid progression of motor impairments indicated a potential risk for an additional involvement of the autonomic nervous system. Yet, there were no episodes of bradycardia or sinus arrest during continuous cardiac monitoring until day 40 when cardiac arrest occurred during tracheal suctioning. Hence, there would have been no clear indication for PM placement until day 40 if EP had not been performed.

Since EP itself is a calculable risk factor for bradycardia, atropine for intravenous application as well as a transcutaneous pacemaker were kept ready for the case of circulatory instability. Cardiac function and blood pressure were continuously monitored. We consider the risk for the patient during EP low and acceptable (taking into account the risks and benefits of this procedure): EP it is a well described procedure with known and treatable risks. On the other hand, if dysautonomia had not been diagnosed and a PM had not been placed, the patient would have been in a critical condition (due to unexpected arrhythmias or sudden cardiac arrest), especially during manoeuvers like tracheal suctioning.

Bradyarrhythmia and asystole as a consequence of vagal overreactivity in GBS are reported with frequencies of about 14% (11). Need of atropine and PM implantation may occur prior to the need of mechanical ventilation (8, 11) and independently of the severity of motor impairments. Hence, even less severe impaired patients should be examined carefully, including repeated EPs.

Transvenous PMs are known to have high complication rates and some authors advise early application of transcutaneous PM on admission day (12). Yet, application of a transcutaneous PM demands deep sedation of the patient as the act of pacing needs high voltage, is painful and potentially traumatizing (13). It is thus reserved for emergencies. As we expected the dysautonomia to resolve, implantation of a permanent pacemaker system was not indicated to avoid possible late complications such as infections or lead defects, especially in a young patient with no structural heart disease. We therefore decided for a transvenous pacing system. Due to the tenacious cardiac dysautonomia that lasted for several weeks, the system was replaced twice prophylactically to avoid infections. There were no complications during long-term application of the temporary pacing system.

## Conclusions

This case illustrates three clinically important aspects: Firstly, symptoms of cardiac dysautonomia can persist over several weeks in GBS. Secondly, the consecutive EP performed during the patient's ICU stay show that this test is a useful and easy to apply bedside test for monitoring vagal dysfunction and that EP might even reflect autonomic recovery in GBS. Thirdly, long-term application of a transvenous temporary pacing system is a feasible way in young GBS patients with tenacious cardiac dysautonomia in order to prevent potential risks of a permanent pacemaker.

## Data Availability Statement

All datasets generated for this study are included in the article/supplementary material.

## Ethics Statement

Ethical review and approval was not required for the study on human participants in accordance with the local legislation and institutional requirements. The patients/participants provided their written informed consent to participate in this study. Written informed consent was obtained from the individual(s) for the publication of any potentially identifiable images or data included in this article.

## Author Contributions

All authors listed have made a substantial, direct and intellectual contribution to the work and approved it for publication.

## Conflict of Interest

The authors declare that the research was conducted in the absence of any commercial or financial relationships that could be construed as a potential conflict of interest.
